# Self-Expandable Metal Stent for Palliation of Dysphagia in Cancer Esophagus at a Tertiary Care Center of North-East India: A Prospective Study

**DOI:** 10.1155/ijso/3331040

**Published:** 2024-12-24

**Authors:** Rohin Kundalia, Revanth Kumar Kodali, Dibyajyoti Deka, Abhijit Talukdar, Deep Jyoti kalita, Gaurav Das, Shivaji Sharma, Mohit Malhotra

**Affiliations:** Department of Surgical Oncology, Dr Bhubaneswar Borooah Cancer Institute, Guwahati, India

**Keywords:** complications, dysphagia, esophageal cancer, nutrition, SEMS

## Abstract

**Background and Aim:** Esophageal cancer is the sixth most common cancer in India with a incidence of around 4.5%. Dysphagia is the primary manifestation of advanced esophageal cancer in 80%–90% of patients. Dysphagia is one of the most distressing and debilitating symptom for the patients. The use of self-expanding metallic stents (SEMS) has revolutionized the treatment of dysphagia in esophageal cancer patients. This study aims to assess the role of SEMS in the palliation of dysphagia in patients with esophageal cancer.

**Methods:** This was a single-center, prospective observational study conducted in the Department of Surgical Oncology at Dr. B. Borooah Cancer Institute in Guwahati, India, from April 2019 to March 2020. Patients were assessed after stent placement for improvements in dysphagia, pain relief, nutritional status, and associated complications.

**Results:** One week after stent placement, 65.3% of patients were able to tolerate semisolid food and 6.1% could tolerate solids. Prior to stent insertion, 87.8% had Grade 4 dysphagia, but at 6 months post-SEMS placement, 90.2% had only Grade 1 dysphagia. This represented a statistically significant improvement in the dysphagia grade, with a p value less than 0.0001. Further analysis using ANOVA and paired t-tests showed significant improvements in weight, body mass index (BMI), and serum albumin at 1, 3, and 6 months after stent placement, with p values less than 0.001.

**Conclusion:** The results of this study demonstrated that the placement of SEMS is a safe and effective palliative intervention for management of dysphagia in carcinoma esophagus, leading to improvements in patient nutrition and quality of life, with relatively few associated complications.

## 1. Introduction

Esophageal cancer is the sixth most common cancer in India with an incidence of around 4.5% in 2018 [1]. Dysphagia is the primary manifestation in 80%–90% of patients with advanced esophageal cancer [2]. Dysphagia is one of the most distressing and debilitating symptoms, as the quality of life for these patients is significantly influenced by their inability to swallow. Therefore, the primary objective of treatment in esophageal cancer patients is the alleviation of dysphagia [3]. Esophageal stenting is done in patients presenting with advanced esophageal cancer who are not suitable for any other treatment modality. There are various types of stents available but self-expanding metal stent (SEMS) insertion is an easy and effective procedure. The placement of SEMS can be associated with complications such as perforation, bleeding, stent migration, tumor ingrowth, and stent occlusion. This study aims to assess the role of SEMS in the palliation of dysphagia in patients with esophageal cancer at a tertiary cancer center of North-East India.

## 2. Materials and Methods

This study is a single-center, prospective observational study conducted in the Department of Surgical Oncology at Dr. B Borooah Cancer Institute in Guwahati over the time period from April 2019 to March 2020.

### 2.1. Aim of the Study

The aim of this study is to assess the role of SEMS in palliating dysphagia for patients with esophageal cancer.

### 2.2. Objectives

The objectives of this study are as follows:

1. To assess the improvement in dysphagia following stenting
2. To assess the improvement in the nutritional status following stenting
3. To investigate the complications associated with stenting in esophageal cancer

### 2.3. Inclusion Criteria

The inclusion criteria include the following:

• Age 18 years or older
• Patients with advanced-stage esophageal cancer who were not candidates for curative treatment and presented with absolute dysphagia
• Patients with malignant airway–esophageal fistulas
• Patients with benign strictures following radiotherapy
• Patients recurring after definitive chemoradiotherapy presenting with dysphagia
• Patients on palliative chemotherapy with absolute dysphagia

### 2.4. Exclusion Criteria

The exclusion criteria include the following:

• Inability to give informed consent
• Patients with other benign causes of dysphagia and esophageal obstruction or stenosis
• Patients with strictures where guide wire was not passable
• Patients with tumors having an upper border located within 4 cm of the cricopharynx

**Figure 1:**
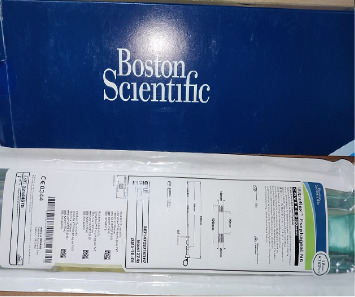
Boston scientific stents were used in all patients (hospital supply).

**Figure 2:**
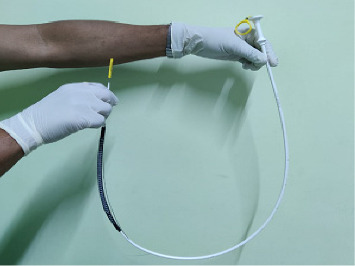
Stent before insertion.

**Figure 3:**
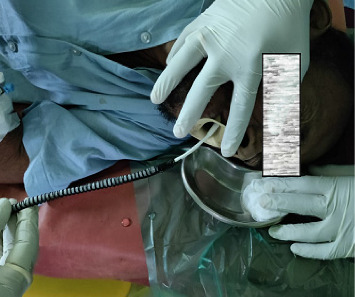
Stent insertion.

**Figure 4:**
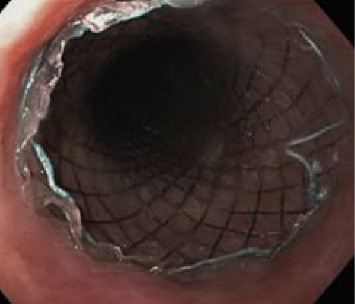
Stent after deployment.

All eligible patients underwent stenting under endoscopic guidance and local anesthesia. The adult gastroscope was passed into the esophagus, across the tumor, and into the stomach in a left lateral position. Once the endoscope reached the stomach, a guidewire was inserted into the antrum, and the scope was slowly pulled back, recording the tumor length and its proximal extension. Stricture dilatation using Savary–Gilliard dilators was performed if necessary, when the endoscopist deemed the stricture to be too tight. We uniformly used Boston scientific stents as they were available in hospital supply (Figures 1 and 2). The esophageal SEMS was then introduced over the guidewire (Figure 3). The gastroscope was reintroduced alongside the stent, and the SEMS was deployed under endoscopic guidance. In all patients, the immediate poststenting deployment was checked by endoscopy, and the in situ stent was showed to the patient and accompanying individuals (Figure 4). If the position was not proper, the stent was pulled up using a grasper for repositioning.

**Figure 5:**
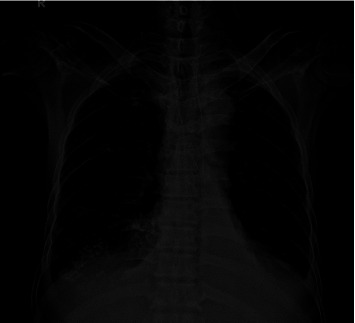
Stent position and expansion in chest X-ray.

Patients were transferred to the general ward following the procedure. Any immediate poststenting complications were documented and appropriately managed. Patients were kept nil per orally with strict bed rest in supine position for 24 h to prevent any stent migration. A postprocedure chest X-ray was performed to confirm stent position and expansion before initiating oral intake (Figure 5). Patients were then gradually advanced from clear liquids to semisolid and solid foods as tolerated, with the timing of each transition being recorded. All patients were discharged only after receiving dietary counseling and instructions on appropriate feeding methods.

All the participants were followed monthly. During each follow-up, their symptoms, performance status, and dysphagia score were assessed. Telephone consultations were conducted for patients who did not attend outpatient department for follow-ups. Dysphagia scores were recorded on Days 3 and 7, and at 1 month, 3 months, and 6 months. Weight, BMI, and albumin levels were measured during follow-up visits at 1 month, 3 months, and 6 months. Any stent-related complications observed during the follow-up period were noted and managed accordingly.

## 3. Results

The clinicopathological characteristics of the study population are shown in Table 1.

In our study, approximately 32% of the population underwent prestenting dilatation using a Savary–Gillard dilator, as they presented with stricturing tumors. Patients were gradually started on oral feeds, ranging from clear liquids to solids, depending on their tolerability. The transition to liquids typically occurred within 2–7 days, while the progression to solid foods typically took 7–15 days (Table 2).

In our study, 12.2% presented with Grade 3 dysphagia and 87.8% presented with Grade 4 dysphagia. After 3 days of stenting, 16.3% had Grade 4 dysphagia, 67.3% had Grade 3, while 16.3% had Grade 2. After 1 week, 65.3% of the population were able to tolerate semisolids and 6.1% were tolerating solids, indicating an improvement in dysphagia. After 1 month, 61.7% population tolerated solids and 36.2% were tolerating semisolids. After 3 months, around 91% of population were having solid diet without difficulty. At the 6th month, similar results were noted. Only one patient had dysphagia progression on third and sixth months due to a tumor in growth into the stent. As these values are computed for statistical analysis using the Chi-square test, there was statistical improvement in dysphagia—*p* value < 0.0001, as shown in Table 3.

Improvement in nutrition was assessed by checking the change in weight, BMI, and albumin levels, pre- and poststenting.

The mean weight, BMI, and albumin levels showed statistically significant improvement at the first, third, and sixth months after stenting, as demonstrated by ANOVA and paired *t*-test analyses (*p* < 0.001) (Tables 4, 5, and 6)

Early c6: Most common early complication noted in our subjects was chest pain which was managed by analgesic. Almost all the patient population experienced mild to moderate pain, 28% had cough, 26% had vomiting, and 8% had postprocedure bleeding (Table 7). One patient had stent migration into the stomach on second postprocedure day which was repositioned endoscopically.

Delayed complications: One individual experienced stent migration at the fifth month, requiring the original stent to be removed and a new one was reinserted. Another patient exhibited worsening dysphagia, which upon evaluation at (Table 7) 5 months was attributed to tumor ingrowth into the stent. This was addressed by placing a new stent.

## 4. Discussion

The northeastern region of India is a major geographical area for esophageal cancer, with particularly high incidence rates observed specifically in the East Khasi Hills district of Meghalaya and the city of Aizawl. The age-adjusted incidence rate is reported to be 75.4 per 1,00,000 in males and 33.6 per 1,00,000 in females in this region [4]. Approximately, 75% of patients present with incurable, advanced disease, or comorbidities that preclude available definitive treatment modalities. These patients require an effective palliative approach which is safe, relatively inexpensive, and easy to implement. Various treatment options exist, such as chemoradiotherapy, direct intratumoral alcohol injection, brachytherapy, photodynamic therapy, thermal ablative procedure such as LASER therapy, and argon beam therapy. These treatment modalities can be associated with either undertreatment, leading to persistent dysphagia or overtreatment, resulting in complications such as perforation [5].

Photodynamic therapy is associated with high cost of photodynamic drugs, cutaneous sensitivity with these drugs, and chances of postprocedure stricture. There may be a need for repeated sessions to maintain the relief achieved [6].

Radiotherapy is not suitable for patients with an esophageal fistula, tumor recurrence after recent radiotherapy, or recurrent dysphagia following initial palliative radiotherapy [7, 8]. Surgical bypass has high morbidity and mortality [9, 10].

Stenting has revolutionized the palliation of dysphagia due to its easy insertion, relatively low complication rates, and reasonably good functional outcomes [11–14]. Hence, in our study, we used stenting as the palliative modality for malignant dysphagia.

Various types of stents are commercially available for dysphagia palliation such as plastic stents and biodegradable stents, but according to a meta-analysis by Yakoub, compared to plastic stents, metal stents are associated with significantly reduced stent-related mortality (1.7% versus 11.1%), reduced esophageal perforation (1.4% versus 9.4%), and lesser stent migration [15]. Silicon-covered stents are useful in palliation of malignant dysphagia as uncovered stents have 13% chances of tumor in growth leading to stent occlusion [16]. Biodegradable stents are useful in palliation of benign strictures; their role in malignant dysphagia is yet to be established. Hence, in our study, we used a partially covered SEMS for palliation.

The success rate of the stenting procedure was around 80%–95%, in most of the studies [15–18], which is comparable to our study which had a procedural success rate of around 95%. Of the 50 patients, two experienced the complication of stent migration—one on the second day and another at the fifth month, requiring reinsertion.

Malignant dysphagia was effectively improved with a prestent mean dysphagia score being 4 and poststent score was two to three on Day 3, 1-2 after 1 week and Grade 1 at 1, 3, 6 months poststenting in around 90% population, and this improvement was statistically significant (*p* < 0.001). This result was comparable to many previous studies that showed a significant improvement in the median dysphagia score after stent insertion [18–20].

The primary aim of treatment in patients with inoperable esophageal cancer is to relieve dysphagia with minimal morbidity and mortality, and thus improve their quality of life. However, in most studies, relief of dysphagia is the only aspect of health-related quality of life being measured. In our study, along with dysphagia improvement, we had also measured the weight, BMI, and albumin levels for assessing the nutritional improvement. Our study found a statistically significant 9%-10% improvement in weight and BMI, as well as a 19% improvement in albumin levels, following stenting. These results confirm that stenting had a positive impact on the nutritional status of our study population.

Stent migration occurred in two patients in our study—one on the second day, requiring repositioning, and another on the fifth month, which necessitated removal of the stent and replacement of a new one. The overall rate of stent migration in our study was only 4%. This finding is consistent with reports by Park et al., indicating that covered SEMS are more prone to migration compared to uncovered SEMS, which aligns with our use of partially covered stents in the present study.

Stent obstruction by tumors was noted in only one patient who was managed by removal and reinsertion of a new stent. Tumor in-growth is a significant problem with uncovered SEMS occurring in 10%–35% of patients [22]. In our study, tumor in-growth occurred only in one patient. In that patient, stent was obstructed by tumor growth both above and below the SEMS, which was documented in other studies also [21–23].

We did not encounter any major complications such as perforation in our study. This can be attributed to the fact that we avoided overdilating the stricture and instead dilated it only to the extent necessary to allow the stent delivery system to effectively navigate across it.

Overall mortality in our study was 18%, but none was either due to procedure-related or stent-related, which was comparable to the published endoscopic series [23].

## 5. Conclusion

This study demonstrates that esophageal stenting using SEMS is a safe and effective palliative intervention for managing malignant dysphagia. The findings indicate that SEMS placement can alleviate symptoms in a relatively short time period, while requiring fewer additional treatments. Furthermore, the procedure was associated with improvements in the nutritional status and quality of life, with only minimal complications observed.

In conclusion, esophageal stenting is a relatively safe and easy procedure to perform. With proper endoscopic training, it can be performed with minimal complications. This intervention quickly alleviates dysphagia in critically ill patients, thus significantly improving their symptoms. Appropriate counseling of the patient regarding the benefits and potential complications of the procedure is essential. Furthermore, educating the patient about dietary habits and other precautions prior to discharge is crucial for a successful outcome. As major and long-term complications are minimal and manageable, esophageal stenting represents one of the most effective ways to enhance the quality of life in terminally ill esophageal cancer patients.

## Figures and Tables

**Table 1:** Clinicopathological characteristics of the study population.

Variable	
Age	32–80 yr median 58 ± 9.2

Gender	Males—31 (62%)
	Females—19 (38%)

Histopathology	Squamous cell carcinoma—45 (90%)
	Adenocarcinoma—5 (10%)

Disease presentation	Posttreatment residual growth—27 (54%)
	Tumor with tracheal infiltration without tracheoesophageal fistula-10 (20%)
	Tracheoesophageal fistula—6 (12%)
	Metastatic disease—6 (12%)
	Recurrent disease—1 (2%)

Stage of the disease	Stage 4—26 (52%)
	Stage 3—24 (48%)

Treatment received	Definitive RT + CT– 17 (34%)
	Palliative care—18 (36%)
	Palliative RT—10 (20%)
	Palliative RT ⟶ palliative CT—3 (6%)
	Definitive RT—2 (4%)

Location of tumor	Upper thoracic—10 (20%)
	Mid thoracic—26 (52%)
	Lower thoracic—14 (28%)

Length of the tumor (cm)	4–14 cm median 7 ± 9.2

**Table 2:** Data showing transition from liquid to solid diet.

.	Starting of liquids (days)	Starting of semisolids (days)	Starting of solids (days)
Mean	4.14	8.24	12.04
Median	4th	9th	12th
Std. deviation	1.291	1.82	1.719
Minimum	2^nd^ day	5^th^ day	7^th^ day
Maximum	7^th^ day	11^th^ day	15^th^ day

**Table 3:** Dysphagia improvement pre- and poststenting.

Dysphagia grade	1	2	3	4	*p* value
Prestenting	0 (0%)	0 (0%)	6 (12.2%)	43 (87.8%)	
Poststenting day 3	0 (0%)	8 (16.3%)	33 (67.3%)	8 (16.3%)	< 0.0001
Day 7	3 (6.1%)	32 (65.3%)	14 (28.6%)	0 (0%)	< 0.0001
1st month	29 (61.7%)	17 (36.2%)	1 (2.1%)	0 (0%)	< 0.0001
3rd month	40 (90.9%)	4 (9.1%)	0 (0%)	0 (0%)	< 0.0001
6th month	37 (90.2%)	2 (4.9%)	1 (2.4%)	1 (2.4%)	< 0.0001

**Table 4:** Comparison of weights pre- and poststenting.

	Prestenting wt	Poststenting wt on 1st month	3rd month	6th month	*p* value
Mean (weight) ± SD	57.18 ± 11.75	58.8 ± 11.82	60.91 ± 12.19	63.06 ± 12.62	< 0.001
Percentage of increase in weight compared to prestenting		2.83%	6.50%	10.30%	

**Table 5:** Comparison of BMI pre- and poststenting.

	Mean (BMI) ± SD	Percentage increase of BMI compared to preprocedure (%)	*p* value
Preprocedure BMI	19.42 ± 2.97		
Poststenting BMI at 1st month	19.99 ± 3	2.9	0.003
3rd month	20.69 ± 3.13	6.5	< 0.001
6th month	21.16 ± 3.81	9.0	< 0.001

**Table 6:** Comparison of albumin.

	Mean (albumin) ± SD	Percentage increase in albumin compared to preprocedure (%)	*p* value
Preprocedure albumin	3.26 ± 0.487		
Poststenting albumin at 1st month	3.48 ± 0.4797	7.0	< 0.001
3rd month	3.7 ± 0.5206	13.5	< 0.001
6th month	3.9 ± 0.5726	19.6	< 0.001

**Table 7:** Early complications.

Complication	Pain	Cough	Vomiting	Bleeding	Stent migration
Number of patients experienced	50 (100%)	14 (28%)	13 (26%)	4 (8%)	1 (2%)

## Data Availability

The data that support the findings of this study are available from the corresponding author upon reasonable request.
